# Development of Korean CARcinogen EXposure: Assessment of the Exposure Intensity of Carcinogens by Industry

**DOI:** 10.1016/j.shaw.2022.05.003

**Published:** 2022-05-23

**Authors:** Dong-Hee Koh, Ju-Hyun Park, Sang-Gil Lee, Hwan-Cheol Kim, Hyejung Jung, Inah Kim, Sangjun Choi, Donguk Park

**Affiliations:** 1 Department of Occupational and Environmental Medicine, International St. Mary's Hospital, Catholic Kwandong University, Incheon, Republic of Korea; 2 Department of Statistics, Dongguk University, Seoul, Republic of Korea; 3 Occupational Safety and Health Research Institute, Korea Occupational Safety and Health Agency, Ulsan, Republic of Korea; 4 Department of Occupational and Environmental Medicine, Inha University, Incheon, Republic of Korea; 5 Department of Occupational and Environmental Medicine, College of Medicine, Hanyang University, Seoul, Republic of Korea; 6 Department of Preventive Medicine, College of Medicine, The Catholic University of Korea, Seoul, Republic of Korea; 7 Department of Environmental Health, Korea National Open University, Seoul, Republic of Korea

**Keywords:** Cancer, Carcinogen, Exposure, Occupational cancer, Occupational exposure

## Abstract

**Background:**

Occupational cancer is a global health issue. The Korean CARcinogen EXposure (K-CAREX), a database of CARcinogen EXposure, was developed for the Korean labor force to estimate the number of workers exposed to carcinogens by industry. The present study aimed to estimate the intensity of exposure to carcinogens by industry, in order to supply complementary information about CARcinogen EXposure intensity to the K-CAREX.

**Methods:**

We used nationwide workplace monitoring data from 2014 to 2016 and selected target carcinogens based on the K-CAREX list. We computed the 95th percentile levels of measurements for each industry by carcinogens. Based on the 95th percentile level relative to the occupational exposure limit, we classified the CARcinogen EXposure intensity into five exposure ratings (1–5) for each industry.

**Results:**

The exposure ratings were estimated for 21 carcinogenic agents in each of the 228 minor industry groups. For example, 3,058 samples were measured for benzene in the manufacturing industry of basic chemicals. This industry was assigned a benzene exposure rating of 3.

**Conclusions:**

We evaluated the CARcinogen EXposure ratings across industries in Korean workers. The results will provide information on the exposure intensity to carcinogens for integration into the K-CAREX. Furthermore, it will aid in prioritizing control efforts and identifying industries of concern.

## 1 Introduction

Occupational cancer is a pertinent global occupational health issue. Currently, one in three individuals in the general population is expected to be diagnosed with any type of cancer when one survives to the age of life expectancy, and one in four individuals in the general population die due to cancers in Korea [1].

The causes of cancer range from genetic to modifiable risk factors, such as smoking and occupation [2,3]. During working hours, workers are exposed to thousands of harmful chemicals and physical and biological agents, and these working conditions can increase the risk of cancer. However, a limited number of agents have been found to be carcinogenic, and most of the other agents have not yet been investigated [4]. Exposure to complex chemical mixtures or co-exposure from multiple sources, such as home, environment, and occupation, further complicate the association between occupational exposure and possible malignancy [5].

Although there are many agents and work conditions to be examined, it is also important to properly utilize the knowledge base that has already been established. Many studies have investigated the carcinogenicity of various agents, such as dust, chemicals, and heavy metals, to prevent occupational cancers. Based on these findings, the International Agency of Research on Cancers (IARC) has developed and currently updated a list of carcinogens, thereby guiding active prevention efforts [6]. However, due to the limited resources, these prevention measures primarily focused on areas where many workers were heavily exposed. Therefore, carcinogen information systems, such as the CARcinogen EXposure (CAREX) have been developed [[7], [8], [9], [10], [11], [12]].

The Korean CAREX (K-CAREX) was recently developed [13], wherein it estimated the exposure prevalence and the number of exposed workers for 20 carcinogens across 228 minor industry groups by referring to three nationwide occupational exposure databases and eliciting the judgment of 37 industrial hygiene experts, targeting the circumstances in 2010.

The present study aimed to develop an estimate of CARcinogen EXposure intensity by industry, using a nationwide workplace monitoring database, which will supply complementary information about CARcinogen EXposure intensity to the K-CAREX. It also describes the estimation procedure of exposure intensity of 21 carcinogenic agents.

## 2 Materials and methods

### 2.1 Data sources

Workplaces with exposure to designated hazardous agents are obliged to periodically monitor the work environment according to a national occupational exposure monitoring system in Korea [14]. Companies requisition work environment monitoring institutions (WEMI), which are private bodies, to monitor the working environment. These results have been compiled electronically by the Korea Occupational Safety and Health Agency (KOSHA) since 2002. The measurement database is known as the work environment measurement database (WEMD) [15,16].

We used the measurement database from 2014 to 2016 to estimate the exposure intensity. The time period is marked by data availability and is chronologically close to the time period of the K-CAREX. This database includes details on industry codes, measurement levels, and sampling time. Air sampling is typically conducted for at least 6 h, according to the guideline (administrative notice). A short-term exposure sampling is also conducted when necessary. The number of samples measured in <4 h (approximately 3.5%) or >10 h (approximately 0.02%) was small. They were regarded as non-routine operations and excluded, along with trivial measurements without appropriate industry codes.

### 2.2 Selection of target carcinogens and definition of carcinogens

Based on the K-CAREX list, we selected 21 carcinogenic agents [13]. We added mists from three strong inorganic acids (hydrochloric acid, nitric acid, and hydrofluoric acid) besides sulfuric acid because they may share a similar carcinogenic mechanism (i.e., low pH) as that of sulfuric acid [17,18]. Workers can be exposed to these strong inorganic acid mists in various industries, including those of plating and semiconductor manufacturing [19]. We excluded three carcinogens (ionizing radiation, ultraviolet radiation, and polycyclic aromatic hydrocarbons) because they were not available in the WEMD.

Arsenic was divided into arsine and arsenic (other than arsine) because the sampling and analytical methods are different for these chemicals. Chromium consisted of inorganic and organic hexavalent chromium, measured by ion chromatography while excluding other compounds, such as metallic chromium measured by atomic absorptiometry (AA). For a nickel, nickel carbonyl was excluded owing to the small number of measurements and different sampling and analytical methods. Crystalline silica consisted of quartz, cristobalite, and tridymite, which were sampled with a cyclone as respirable dust.

Accordingly, arsenic, arsine, asbestos, benzene, beryllium, 1,3-butadiene, cadmium (Cd), hexavalent chromium (Cr6+), ethylene oxide (EtO), formaldehyde, hydrochloric acid (HCl), hydrofluoric acid (HF), mineral oil mist, nickel (Ni), nitric acid, crystalline silica, sulfuric acid, trichloroethylene (TCE), vinyl chloride monomer (VCM), welding fumes, and wood dust were selected as target carcinogens.

### 2.3 Standard industrial classification

The WEMD classifies industries according to the Korean Standard Industrial Classification (KSIC-9) based on the International Standard Industrial Classification (ISIC, 4th revision). The three-digit minor industry code of the ISIC was used as the standard industrial classification (SIC) in our study. The industry code is assigned by industrial hygienists who conduct workplace monitoring. Industrial hygienists commonly refer to industry names on the certificate for business registration of monitored companies.

### 2.4 Data cleaning and treatment

To ensure uniformity among the 160 WEMIs that sample and report workplace exposure, a quality control program for sampling and analytical methods is performed periodically by the KOSHA [20]. Despite the active quality control program, analytical institutions have different equipment and analytical settings, resulting in varied results.

The limit of detection (LOD) values is particularly variant when it comes to different analytical institutions. However, the WEMD contains no information about LOD values; hence, we obtained reporting LOD levels from several WEMIs. Based on the reporting LODs, we reached a consensus on a single LOD for each carcinogen, basically averaging LOD levels from these analytical institutions (Table 1).

**Table 1:** Sampling and analytical method, reporting limit of detection, and occupational exposure limit of carcinogens

Name	Sampling media	Flow rate (L/min)	Sampling time (min)	Analytical equipment	LOD	OEL
Arsine	Charcoal tube	0.02	360	AA-GF	0.0001 ppm	0.005 ppm
Arsenic	MCE filter	1	360	AA-GF	0.03 μg/m^3^	0.01 mg/m^3^
Asbestos	MCE filter	2	360	PCM	0.003 fiber/cc	0.1 fiber/cc
Benzene	Charcoal tube	0.03	360	GC	0.03 ppm	0.5 ppm
Beryllium	MCE filter	2	360	ICP	0.002 μg/m^3^	0.002 mg/m^3^
1,3-Butadiene	Charcoal coated with TBC	0.05	360	GC	0.006 ppm	2 ppm
Cd	MCE filter	2	360	AA	0.2 μg/m^3^	0.01 mg/m^3^
Cr6+	PVC filter	2	360	IC	0.2 μg/m^3^	Based on 0.01 mg/m^3^ (non-soluble); 0.05 mg/m^3^ (soluble)
EtO	HBr-coated carbon beads, 100 mg/50 mg	0.5	360	GC-ECD	0.00003 ppm	1 ppm
Formaldehyde	Cartridge containing silica gel coated with 2,4-dinitrophenylhydrazine	0.5	360	HPLC	0.001 ppm	0.3 ppm
HCl	Silica-gel tube	0.2	360	IC	0.002 ppm	1 ppm
HF	Silica-gel tube	0.2	360	IC	0.002 ppm	0.5 ppm
Mineral oil mist	PTFE filter	2	360	Gravimetric	0.01 mg/m^3^	0.8 mg/m^3^
Ni	MCE filter	2	360	AA	0.08 μg/m^3^	Based on 0.1 mg/m^3^ (soluble); 0.2 mg/m^3^ (non-soluble); 1.5 mg/m^3^ (metal)
Nitric acid	Silica-gel tube	0.2	360	IC	0.002 ppm	2 ppm
Silica, crystalline	PVC filter. Cyclone	1.7	360	FTIR	0.3 μg/m^3^	0.05 mg/m^3^
Sulfuric acid	Silica-gel tube	0.2	360	IC	0.03 mg/m^3^	0.2 mg/m^3^
TCE	Charcoal tube	0.03	360	GC	0.09 ppm	Based on 10 ppm (2016); 50 ppm (2014–2015)
VCM	Tandem charcoal tubes	0.05	360	GC	0.003 ppm	1 ppm
Welding fume	MCE filter	2	360	Gravimetric	0.01 mg/m^3^	5 mg/m^3^
Wood dust	IOM sampler	2	360	Gravimetric	0.01 mg/m^3^	Based on 1 mg/m^3^ (others); 0.5 mg/m^3^ (red cedar)

Abbreviations: LOD, limit of detection; OEL, occupational exposure limit; Cd, cadmium; Cr6+, hexavalent chromium; EtO, ethylene oxide; HCl, hydrochloric acid; HF, hydrofluoric acid; Ni, nickel; TCE, trichloroethylene; VCM, vinyl chloride monomer; AA-GF, atomic absorptiometry-graphite furnace; PCM, phase contrast microscopes; GC, gas chromatography; ICP, inductively coupled plasma; AA, atomic absorptiometry; IC, ion chromatography; GC-ECD, gas chromatography-electron capture detector; HPLC, high-performance liquid chromatography; FTIR, Fourier transform infrared.

In the WEMD, a large proportion of measurements showed extremely low to zero (not detected) levels. Therefore, values below the LOD were treated as censored and replaced with half of the LOD [21]. Different occupational exposure limits (OELs) exist for different carcinogen compounds. For instance, the OEL of non-soluble hexavalent chromium was 0.01 mg/m^3^, whereas that of soluble hexavalent chromium was 0.05 mg/m^3^. We chose 0.01 mg/m^3^ as a representative OEL for Cr6+ for computational purposes. Likewise, 0.1 mg/m^3^ and 1 mg/m^3^ were chosen for nickel and wood dust, respectively (Table 1). The OEL of TCE in Korea decreased from 50 to 10 ppm in 2016; we chose 10 ppm as the representative OEL for the TCE.

### 2.5 Statistical analysis

In a previous pilot study using the WEMD, we computed summary statistics, including mean, geometric mean, and X95 values for both airborne and blood lead, and then examined optimal exposure intensity indicators by comparing airborne measurements with blood lead measurements [22]. We concluded that the mean and X95 values would be optimal exposure intensity indicators for the WEMD based on the results of rank correlation analyses. Furthermore, X95 showed a better correlation than the mean when restricting industries to those with 20 or more measurements.

In line with the previous pilot study, the present study first calculated X95 for each three-digit SIC and then computed the exposure ratings based on the X95 level compared to the corresponding OEL [23]. The X95 value has been used for initial exposure assessment using the concept of “major/minor” cuts. Any exposure scenario may be characterized as “minor” if the anticipated upper-end exposure is <1/10th of the OEL, which would be considered “acceptable” [23]. Furthermore, the exposure ratings have been used for categorizing risks and management for a similar exposure group (SEG) based on an estimate of the X95 relative to the OEL [23]. We adopted the same scheme to assign exposure intensity (1–5 categories) to three-digit minor industries in the present study (Table 2). In addition, we have added the “0” category (not rated) to the scheme for industries with <20 measurements to alleviate potential bias due to small samples because of industry code errors [22].

**Table 2:** Exposure rating scheme based on decision statistics of 95^th^ percentile (X95)

Exposure rating	Definition
0	Not rated; <20 measurements
1	X95 < 1% of OEL
2	X95 < 10% of OEL
3	X95 within10–50% of OEL
4	X95 within 50–100% of OEL
5	X95 > 100% of OEL

Abbreviations: OEL, occupational exposure limit.

The distributions of carcinogens with low censoring rates, such as welding fume (3%) approximated log-normal distributions across industries (graphs not shown), whereas other carcinogens with high censoring rates, such as arsine (99%), could not be examined for distributions due to high censoring rates. We assumed that all of the carcinogens would follow lognormal distributions.

**Fig. 1:**
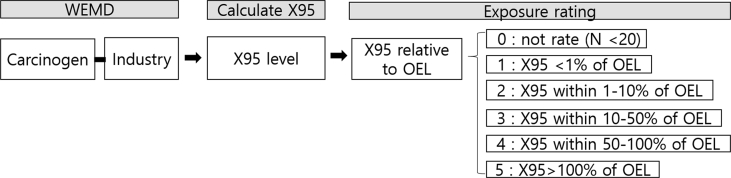
Schematic diagram of the overall estimation process of exposure intensity. Note: WEMD, work environment measurement database; X95, 95th percentile; *N*, number of measurements; OEL, occupational exposure limit.

The overall estimation process of exposure intensity is illustrated schematically in Fig. 1.

## 3 Results

The total number of measurements, censoring rate, and distribution of exposure ratings for each carcinogen by industry are presented in Table 3. Welding fume had the largest number of measurements (190,576) followed by nickel (148,728) and mineral oil mist (136,027). Arsine showed the highest censoring rate of below the LOD (99%), while welding fume showed the lowest censoring rate (3%). A total of 19,661 benzene measurements are included in the WEMD between 2014 and 2016, and the highest exposure rating is 4, which is assigned to seven three-digit minor industry groups (Table 3).

**Table 3:** Censoring rate by carcinogen, and distributions of exposure ratings by carcinogen and industry (total 228 minor industries)

Carcinogen	Censoring rate			Number of three-digit industry by exposure ratings					
	Censored	Total	Rate (%)	0 (not rated)	1	2	3	4	5
Arsine	1,301	1,311	99	223	0	5	0	0	0
Arsenic	2,208	2,697	82	209	11	3	3	1	1
Asbestos	411	652	63	219	0	5	3	0	1
Benzene	17,995	19,661	92	156	0	35	30	7	0
Beryllium	297	317	94	224	2	1	1	0	0
1,3-Butadiene	4,532	5,048	90	206	11	8	1	1	1
Cd	6,553	7,494	87	176	0	38	12	2	0
Cr6+	31,974	40,513	79	130	0	62	29	6	1
EtO	6,836	11,443	60	211	2	3	10	1	1
Formaldehyde	14,414	51,631	28	144	1	17	55	11	0
HCl	42,932	63,502	68	110	7	107	4	0	0
HF	15,196	17,636	86	177	17	27	6	1	0
Mineral oil mist	19,925	136,027	15	141	0	0	82	5	0
Ni	66,093	148,728	44	107	10	96	14	1	0
Nitric acid	30,880	45,667	68	128	46	54	0	0	0
Silica, crystalline	33,882	53,974	63	142	7	18	51	8	2
Sulfuric acid	81,959	88,999	92	102	0	48	77	1	0
TCE	9,220	18,295	50	154	10	13	18	11	22
VCM	2,149	2,814	76	210	4	6	7	1	0
Welding fume	5,997	190,576	3	108	0	1	109	8	2
Wood dust	1,282	22,739	6	164	0	0	15	47	2

Note: Censored, values below the limit of detection; Cd, cadmium; Cr6+, hexavalent chromium; EtO, ethylene oxide; HCl, hydrochloric acid; HF, hydrofluoric acid; Ni, nickel; TCE, trichloroethylene; VCM, vinyl chloride monomer.

Table 4 presents the number of measurements, censoring rate, and exposure ratings ranked among the top 20 industries for benzene exposure. For instance, the manufacturing of basic chemicals (201) industry contained 3,058 benzene measurements and showed a 92% censoring rate, in which exposure was rated 3. The number of the three-digit minor industry that was assigned exposure rating 4 was seven. Detailed exposure intensity results for 21 carcinogenic agents across 228 minor industries are available online at https://koreancarex.shinyapps.io/k-carex_intensity/. In addition, we presented exposure ratings of all industries, including industries with <20 samples in Supplemental Table 1.

**Table 4:** Censoring rate and exposure rating of benzene by industry (top 20 industries based on exposure rating)

SIC	Explanation	Censoring rate			Exposure rating
		Censored	Total	Rate (%)	
181	Printing and service activities related to printing	78	104	75	4
221	Manufacture of rubber products	135	153	88	4
222	Manufacture of plastic products	123	206	60	4
251	Manufacture of structural metal products, tanks, reservoirs, and steam generators	163	217	75	4
259	Manufacture of other metal products; metal working service activities	269	357	75	4
320	Manufacture of furniture	61	87	70	4
949	Other membership organizations	26	28	93	4
107	Manufacture of other food products	144	155	93	3
152	Manufacture of footwear and parts of footwear	54	71	76	3
162	Manufacture of wood products	61	85	72	3
201	Manufacture of basic chemicals	2,808	3,058	92	3
203	Manufacture of synthetic rubber and of plastics in primary forms	751	896	84	3
204	Manufacture of other chemical products	1,800	1,986	91	3
212	Manufacture of medicaments	540	581	93	3
243	Cast of metals	87	106	82	3
262	Manufacture of electronic components	67	73	92	3
283	Manufacture of insulated wires and cables, including insulated code sets	29	34	85	3
291	Manufacture of general-purpose machinery	172	214	80	3
292	Manufacture of special-purpose machinery	229	272	84	3
302	Manufacture of bodies for motor vehicles; manufacture of trailers and semitrailers	18	25	72	3

Note: Censored, values below the limit of detection.

Table 5 shows the number of measurements, censoring rate, and exposure rating of each carcinogenic agent for the “manufacture of basic chemicals (201)” industry, as an example. A total of 21 agents were measured in this industry, including 3,058 benzene measurements. Benzene showed a 92% censoring rate with an exposure rating of 3. Arsine was measured in this industry, but the number of measurements was <20; therefore, the exposure rating was assigned “0” (not rated).

**Table 5:** Censoring rate and exposure rating (1–5) by carcinogen for the “manufacture of basic chemicals (201)” industry

Carcinogen	Censoring rate			Exposure rating
	Censored	Total	Rate (%)	
Arsine	10	11	91	0 (not rated)
Arsenic	21	21	100	1
Asbestos	0	0	NA	0
Benzene	2,808	3,058	92	3
Beryllium	0	0	NA	0
1,3-Butadiene	677	798	85	2
Cd	136	182	75	3
Cr6+	608	710	86	3
EtO	270	340	79	3
Formaldehyde	380	963	39	3
HCl	2,959	4,195	71	2
HF	671	828	81	3
Mineral oil mist	28	147	19	3
Ni	847	1,740	49	2
Nitric acid	1,394	1,869	75	1
Silica, crystalline	574	740	78	3
Sulfuric acid	4,507	4,917	92	3
TCE	143	149	96	1
VCM	228	271	84	3
Welding fume	16	942	2	3
Wood dust	0	14	0	0

Note: Censored, values below the limit of detection; Cd, cadmium; Cr6+, hexavalent chromium; EtO, ethylene oxide; HCl, hydrochloric acid; HF, hydrofluoric acid; Ni, nickel; TCE, trichloroethylene; VCM, vinyl chloride monomer; NA, not applicable.

## 4 Discussion

In this study, we estimated the exposure intensity of 21 carcinogenic agents across 228 minor industries using a nationwide exposure measurement database using a previously tested intensity indicator for exposure intensity development [22]. The results will provide information on the exposure intensity of carcinogens as a complement to the previously developed K-CAREX [13].

We assessed the exposure intensity for 21 carcinogenic agents selected, based on the K-CAREX carcinogens list [13]. Workers are exposed to arsenic in many industries, including the “basic precious and non-metal (242)” industries [24]. However, some measurements were taken from an industry where arsenic exposure was unlikely to occur, such as the “manufacturing of other food products (107).” When we further investigated the measurement information, we found that several companies run laboratories in which arsenic was used. Most food-producing companies do not use arsenic; therefore, it should be considered that the exposure ratings only apply in certain circumstances where actual exposure occurs in the industry.

Asbestos exposure is usually associated with construction, shipbuilding, and steel foundry [25]. Asbestos was widely used as an insulating material, and some remnants still persist, although most of them have been abated. For instance, a chemical plant may cover the asbestos remnants with paste to prevent weathering of asbestos materials if the asbestos-containing materials cannot be removed [26]. Therefore, this chemical plant is not obliged to measure airborne asbestos during periodic work environment monitoring; therefore, there is no asbestos measurement presented in the “manufacturing of basic chemicals (201)” industry, as shown in Table 5. However, asbestos exposure can occur during maintenance operations. Maintenance operations in petrochemical plants were mainly conducted by maintenance workers employed by companies specializing in these operations [27]. These maintenance companies may be classified as “architectural, engineering, and related technical services (721).” This complex contract and subcontract structure may lead to confusion when interpreting exposure intensity ratings.

The Korean OEL for beryllium is 2 μg/m^3^; however, this cannot protect workers from contracting chronic beryllium disease (CBD) or beryllium sensitization [28]. Therefore, the threshold limit value (TLV) of beryllium according to the American Conference of Governmental Industrial Hygienists (ACGIH) has been reduced to 0.05 μg/m^3^. Similarly, the permissible exposure limit of the US Occupational Safety and Health Administration (US OSHA) has been reduced to 0.2 μg/m^3^, which is far lower than that of the Korean OEL. This difference in the OELs should be considered when using the current exposure ratings for other health effects, such as CBD.

Basic chemicals, such as benzene and 1,3-butadiene, can be highly exposed during facility maintenance operations rather than during ordinary manufacturing processes [29,30]. Workplace monitoring is usually conducted for 6 h during normal manufacturing processes; however, if necessary, short-term sampling is also performed. Maintenance operations in petrochemical plants would be one such case. Approximately 2% of benzene samples and 3% of 1,3-butadiene samples in the WEMD were short-term samples, which showed much higher levels than samples measured at 6 h (data not shown). In the present study, we removed the short-term samples to account for the different sampling frameworks; therefore, our results do not reflect short-term, temporarily high exposure circumstances.

In 1,3-butadiene, the manufacturing of general-purpose machinery showed an exposure rating of 5, although exposure to 1,3-butadiene is unlikely to occur in the machinery manufacturing process [31]. We examined the data in detail and found that the measurements from one company showed very high 1,3-butadiene levels. Although the industry was classified as a machinery manufacturing industry, the work process implied that the samples were taken from petrochemical plants or refineries during the maintenance or installation of facilities. Thus, in some cases, the industry where exposures occur is more critical than the work circumstances of the original industry. Moreover, potential confusion from these uncommon working conditions should be considered, especially when unlikely exposures are detected.

The Korean OEL of the TCE changed from 50 to 10 ppm in 2016. We chose 10 ppm as the representative OEL to compute the exposure ratings. The mean TCE in 2016 was lower than that in 2014 and 2015 (data not shown). Owing to the change effect in OEL in 2016 and the reduced OEL application, many industries showed higher exposure ratings for TCE than for other carcinogenic agents. Therefore, the exposure ratings of TCE should be interpreted with this change in mind.

In a previous study, we calculated the summary statistics of airborne lead measurements and compared them with those from blood lead data [22]. The results indicated that X95 is likely to be an optimal indicator when restricting results to industries containing ≥20 measurements. The result supports our current findings, which were obtained using the X95 to estimate exposure ratings. However, care should also be exercised when extending this conclusion to other carcinogenic agents.

For several carcinogenic agents, such as arsine, asbestos, benzene, Cd, Cr6+, mineral oil mist, sulfuric acid, welding fumes, and wood dust, the lowest exposure rating was 2, because the LODs were >1% of the OELs in these carcinogens. The LOD may vary according to batches and institutions; however, the WEMD contains no information on LODs. To address the issue, we contacted experienced analysts in several WEMIs and obtained the reporting LODs of the WEMIs. Then, we reached a final single LOD for each carcinogen, basically averaging the reporting LODs. All measuring institutions periodically participate in quality control programs for the performance of analysis according to the standard sampling and analytical methods of the KOSHA, which is similar to those of the US National Institute of Occupational Safety and Health (NIOSH) [20,22]. Furthermore, the essential analysis equipment (e.g., gas chromatography, AA) of the measuring institution is specified by the guideline (administrative notice), and the measurement time is stipulated to be at least 6 h. Therefore, it is considered reasonable to apply the average value of LOD of some measuring institutions to this study. However, applying a single LOD might affect the exposure ratings.

We have added the “0” category (not rated) to the scheme for industries with <20 measurements. However, restricting the industry to ≥20 measurements may lose sensitivity to detect minor exposure circumstances while increasing the specificity of the exposure.

Current estimates of exposure intensity are different from those of other occupational exposure information systems, such as the Finnish Job-Exposure Matrix (FINJEM) [32] and Canadian CAREX [11]. The estimate of exposure intensity of the K-CAREX is an ordinal scale, similar to the Canadian CAREX, unlike the continuous scale of the FINJEM. The FINJEM covers decades of time periods, whereas the K-CAREX and Canadian CAREX are set at the time of generation. The WEMD is not publicly available; therefore, we were unable to provide summary statistics such as mean values. However, we are planning to update the K-CAREX as workplace monitoring data accumulate. An occupation-based exposure matrix such as the FINJEM is useful for exposure assessment tools in occupational epidemiology and hazard surveillance tools. Currently, the WEMD contains no data on job information; therefore, it is necessary to incorporate job information in the occupational exposure and health surveillance systems of Korea in the future.

The strength of this study is depicted in the ability to assess CARcinogen EXposure intensity across a wide range of industries. However, caution is essential when interpreting the results due to the limitations of the data source and analytical methods. First, we assessed exposure according to industry; however, it will not account for variabilities among processes and sites [33]. The estimate of exposure intensity of an industry does not apply to all processes and sites in the industry. Second, the exposure rating scheme used for SEGs [23] was adopted to assign exposure intensity according to industry. Therefore, direct result interpretation as to “major/minor” cut and “applicable management/controls” may not be applicable. Third, some carcinogens showed a high censoring rate (e.g., arsine 99%). Semiconductor factories conduct mandatory arsine monitoring periodically, which will result in a high proportion of measurements below the LOD because arsine gas may be detected only in abnormal conditions, such as leakage [19]. Therefore, the high censoring rate of an industry may not imply that the industry is safe all the time. Fourth, the estimates of exposure ratings are assigned to carcinogens but not to ordinary chemicals. CARcinogen EXposure should be decreased as much as possible [34]; therefore, the estimates of exposure ratings may not endorse safety in terms of cancer risks. Fifth, we used the Korean OEL between 2014 and 2016 as a reference OEL. The Korean OEL mainly refers to TLVs of the ACGIH [35]. Applying different OELs would result in different exposure ratings.

Our study also has a few limitations stemming from the characteristics of workplace exposure monitoring, as described in previous studies [15,22]. First, workplace monitoring is conducted based on the maximum risk rather than a random sampling of participants. Second, workplace monitoring is performed by private WEMIs, and the companies pay the fees. This payment structure may affect monitoring results because companies are usually concerned about the disadvantages of violations of OELs. Third, small companies may be under-represented rather than large companies because of monitoring fees or ignorance.

In conclusion, we estimated the exposure intensity for 21 carcinogenic agents across 228 minor industrial groups using a nationwide workplace monitoring database. The study results will supply complementary information about CARcinogen EXposure intensity to the K-CAREX. Furthermore, it will aid in prioritizing prevention efforts for occupational cancers and identifying industries of concern for additional monitoring.

## Ethics

The study protocol was reviewed and approved by the Institutional Review Board of the Catholic Kwandong University, International St. Mary's Hospital, Incheon, Republic of Korea (IS17QIMI0035).

## Conflict of Interest

The authors declare that they have no conflicts of interest.
